# Accumbens dopamine D2 receptors increase motivation by decreasing inhibitory transmission to the ventral pallidum

**DOI:** 10.1038/s41467-018-03272-2

**Published:** 2018-03-14

**Authors:** Eduardo F. Gallo, Jozsef Meszaros, Jeremy D. Sherman, Muhammad O. Chohan, Eric Teboul, Claire S. Choi, Holly Moore, Jonathan A. Javitch, Christoph Kellendonk

**Affiliations:** 1 Department of Psychiatry, College of Physicians and Surgeons, Columbia University, New York, NY 10032 USA; 2 Division of Molecular Therapeutics, New York State Psychiatric Institute, New York, NY 10032 USA; 3 Division of Integrative Neuroscience, New York State Psychiatric Institute, New York, NY 10032 USA; 4 Department of Pharmacology, College of Physicians and Surgeons, Columbia University, New York, NY 10032 USA

## Abstract

Dopamine D2 receptors (D2Rs) in the nucleus accumbens (NAc) regulate motivated behavior, but the underlying neurobiological mechanisms remain unresolved. Here, we show that selective upregulation of D2Rs in the indirect pathway of the adult NAc enhances the willingness to work for food. Mechanistic studies in brain slices reveal that D2R upregulation attenuates inhibitory transmission at two main output projections of the indirect pathway, the classical long-range projections to the ventral pallidum (VP), as well as local collaterals to direct pathway medium spiny neurons. In vivo physiology confirms the reduction in indirect pathway inhibitory transmission to the VP, and inhibition of indirect pathway terminals to VP is sufficient to enhance motivation. In contrast, D2R upregulation in the indirect pathway does not disinhibit neuronal activity of the direct pathway in vivo. These data suggest that D2Rs in ventral striatal projection neurons promote motivation by weakening the canonical output to the ventral pallidum.

## Abstract (web summary)

Dopamine D2 receptor activity in the nucleus accumbens is associated with regulation of motivated responding. Here the authors show that overexpression of D2 receptors specifically in ventral striatal projection neurons leads to an increase in the willingness to work by reducing inhibitory transmission to ventral pallidal neurons.

## Introduction

Motivational abnormalities are observed in psychiatric disorders including depression, schizophrenia, attention-deficit hyperactivity disorder (ADHD) and substance abuse. A core deficit of these disorders, motivational dysfunction is disruptive to everyday life and in many cases cannot be treated^1,2^.

The nucleus accumbens (NAc) and its dopaminergic innervation are particularly critical for motivated behavior, and pharmacological studies implicate striatal dopamine receptors in the regulation of incentive motivation^3,4^. Antagonists for D1-like and D2-like receptors delivered to the NAc decrease the willingness to work for rewards^5^. Rather than affecting the hedonic value of the reward, it has been suggested that NAc dopamine promotes behavioral activation by regulating effort-related processes and overcoming work-related response costs^3,6–8^. In line with these observations in animal models, abnormalities in striatal D2 receptor (D2R) levels have been consistently observed in human disorders with altered motivation including addiction, schizophrenia and ADHD^9–16^. Moreover, higher D2R binding in left striatum relative to the right striatum has been associated with higher incentive motivation^17^.

In a recent behavioral study, we demonstrated that overexpression of postsynaptic D2Rs in the mouse NAc increased motivation without altering consummatory behavior, the representation of the value of the reinforcer, or the ability to flexibly use reward-associated cues^18^. Yet, the cellular and neurophysiological mechanisms by which dopamine D2Rs regulate motivated behavior remain unclear. A critical barrier in the study of D2R function in the NAc has been that D2Rs are expressed in multiple cell types including medium spiny neurons (MSNs), cholinergic interneurons (CINs), as well as on terminals of dopaminergic neurons. Therefore, strategies that enable selective manipulation of D2Rs in relevant cell populations are uniquely suited to identifying cell-specific roles of D2Rs in motivation and to interrogate the consequences of D2R function on basal ganglia circuitry underlying motivated behavior. For this purpose, we recently developed an adeno-associated virus (AAV)-based approach that, in contrast to our initial study^18^, allows selective overexpression of D2Rs in the striatal output pathway of the adult NAc core that endogenously express D2Rs, the indirect pathway (D2R-OE_NAcInd_ mice)^19^.

The indirect pathway is one of the two main output pathways of the striatum. The indirect pathway expresses D2Rs and modulates the internal segment of the globus pallidus (GPi) and the substantia nigra pars reticulata (SNr) output nuclei through a polysynaptic circuit via the external segment of the GP (GPe)^20^. In contrast, the direct pathway predominantly expresses D1Rs and not D2Rs. It projects monosynaptically to the basal ganglia output nuclei of the GPi and the SNr, but also innervates the “indirect” GPe via bridging collaterals^21,22^. The direct and indirect pathways exert opposing effects on thalamo-cortical activation and are therefore often referred to as “Go” and “NoGo” pathway, respectively. This opposing function has been historically studied in the context of motor regulation and Parkinson’s disease^23^. Within the dorsomedial striatum, artificial stimulation of the direct pathway promotes locomotion, whereas activation of the indirect pathway inhibits locomotion^8,22,24^. Nevertheless, both pathways are activated during movement initiation^25^.

Within the ventral striatum, analogous direct and indirect pathways have also been described^26–28^. Like dorsal striatal D2R-expressing MSNs (D2-MSNs) that innervate the GPe, NAc D2-MSNs project to the ventral pallidum (VP). However, recent work suggests that the ventral striatal output pathways are less segregated than those of the dorsal striatum since ventral striatal D1-expressing MSNs (D1-MSNs) of the “direct” pathway robustly innervate the VP, in contrast to the less extensive innervation of the GPe by dorsal striatal D1-MSNs^29^. Despite this relative difference in architecture, opposing functions of D1R versus D2R-expressing ventral striatal pathways have been described. For example, while NAc D1-MSNs have been shown to promote psychostimulant sensitization, conditioned place preference, and self-administration, D2-MSNs of the indirect pathway generally inhibit these behaviors^30–33^. Thus, we also refer to D1-MSNs and D2-MSNs in the NAc as direct pathway and indirect pathway neurons, respectively.

In dopamine neurons, presynaptic D2-autoreceptors are thought to inhibit dopamine release via G_βγ_-mediated inhibition of voltage-gated calcium channels and activation of Kv1.2 potassium channels^34–36^. Likewise, slice physiology studies have shown that pharmacological activation of D2Rs reduces inhibitory transmission at the two main outputs of the indirect pathway, intra-striatal collaterals and striatopallidal synapses^37–40^.

Here, we find that selective upregulation of D2Rs in the indirect pathway of the NAc enhances the willingness to work for food in a progressive ratio (PR) task. Using in vitro slice recordings, we find that this increase in motivation is associated with decreased inhibitory transmission from D2-MSNs, resulting in decreased collateral inhibition of direct pathway MSNs within the NAc and in blunted indirect pathway output from the NAc to the VP. However, despite a pronounced decrease in collateral inhibition in the slice after D2-MSN stimulation, D1-MSNs are not more active during the PR task than in control mice, suggesting that collateral inhibition during the PR task may not be strong enough to influence direct pathway activity. In contrast, in vivo recordings of VP activity following cortical stimulation reveals a decrease in VP inhibition upon D2R upregulation. Moreover, using pathway-specific expression of a designer receptor in vivo, we show that inhibition of synaptic transmission from the indirect pathway to the VP is sufficient to enhance motivated behavior. These findings suggest that D2Rs in NAc D2-MSNs regulate motivated behavior by disinhibiting the ventral pallidum.

## Results

### D2R upregulation in NAc D2-MSNs increases motivation

**Fig. 1:**
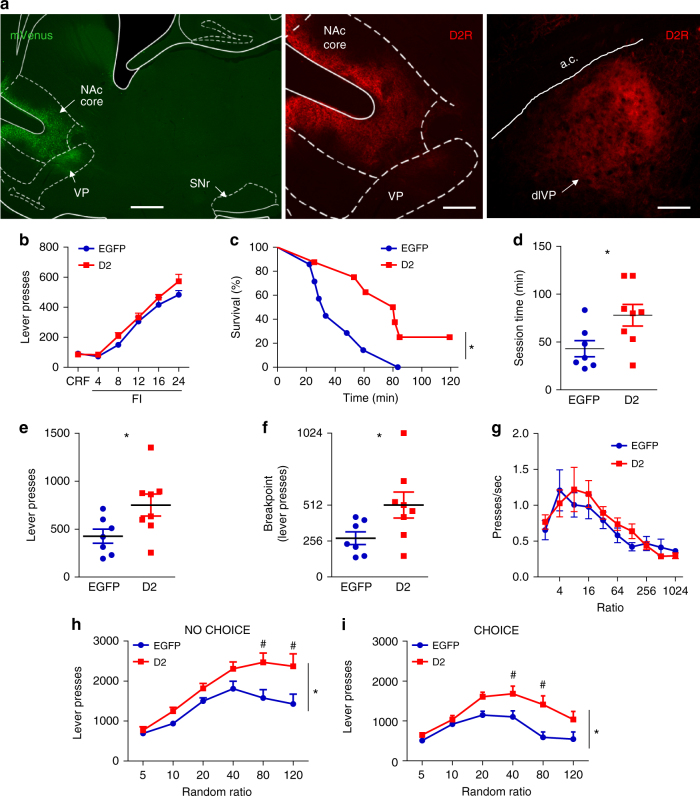
D2R upregulation enhances motivated behavior. **a** (Left) Sagittal image of a *Drd2-Cre* mouse showing that virus-mediated expression (mVenus) is largely restricted to neurons in the NAc core. mVenus-positive terminal fields are observed in the VP, but not the SNr. Scale = 500 µm. (Middle) Higher magnification image shows increased D2R immunolabeling in the NAc core and in the VP. Scale = 250 µm. (Right) Coronal view of viral D2R-positive terminal fields in the dorsolateral VP (dlVP). Scale = 150 µm. **b** D2R overexpression did not alter operant learning on continuous reinforcement (CRF) schedules or performance on fixed interval (FI) schedules conducted prior to progressive ratio (PR) testing. **c** Kaplan–Meier survival function showing the percentage of mice that continues to respond on the PR schedule as a function of session time. D2R-OE_NAcInd_ mice continue to respond significantly longer than control EGFP_NAcInd_ mice. Average data of three different PR sessions are shown (*n* = 7 or 8 mice/group). **d** Total session duration was significantly increased for D2R-OE_NAcInd mice_. **e**, **f** D2R-OE_NAcInd_ exhibit significantly increased lever presses and a higher breakpoint compared to controls. **g** Press rate, plotted here as a function of the ratio requirement, was unaltered. **h** In a random ratio (RR) schedule without concurrent food presentation (NO CHOICE), D2R-OE_NAcInd_ mice showed significantly higher lever-pressing than controls (*n* = 7 mice/group). **i** When a freely-available, standard chow was simultaneously presented (CHOICE), D2R-OE_NAcInd_ mice continued to press significantly more than controls. Error bars = s.e.m.

We selectively targeted NAc D2-MSNs by bilaterally injecting Cre-dependent adeno-associated viruses (AAVs) expressing either D2R-IRES-mVenus or EGFP into the NAc core of *Drd2-Cre* mice. We previously demonstrated that this strategy results in a three-fold increase in D2R radioligand binding in NAc membranes^19^. Upregulation was essentially restricted to D2-MSNs, with very limited transgene expression in cholinergic interneurons (1 out of 20 ChAT + neurons)^19^. We further observed that viral-mediated D2R expression was specific to the indirect projection pathway, as mVenus or D2R immunoreactive terminals were observed exclusively in the projection target of NAc D2-MSNs, the VP, but not in the substantia nigra pars reticulata (Fig. 1a).

To assess the effect of D2R upregulation on the willingness of mice to work for a food reward, we used a progressive ratio (PR) task, in which the lever press requirement doubled with each successive reward earned. Mice expressing EGFP (EGFP_NAcInd_) or overexpressing D2R (D2R-OE_NAcInd_) in D2-MSNs underwent operant training 4 weeks after surgery using a continuous reinforcement (CRF) schedule, followed by fixed interval (FI) training prior to PR testing. The groups did not differ in performance on either CRF (*t* = 0.68, *p* = 0.51) or on any FI schedule tested (Fig. 1b, all ps > 0.05), suggesting that D2R upregulation does not impact operant training.

In the PR task, a survival analysis of the percentage of mice that continue to respond as a function of time revealed that, compared to EGFP_NAcInd_ controls, D2R-OE_NAcInd_ mice responded for a significantly longer time (*χ*^2^ = 4.631, *p* < 0.05, Log-rank test; Fig. 1c, d). Moreover, D2R-OE_NAcInd_ mice showed significantly increased lever pressing (*t* = 2.309, *p* < 0.05, *n* = 7–8/group) and a higher breakpoint (*t* = 2.188, *p* < 0.05, *n* = 7–8/group; Fig. 1e, f). In contrast, the press rate was not altered in D2R-OE_NAcInd_ mice (Fig. 1g).

To determine whether the small subset of cholinergic interneurons that overexpress D2Rs in the *Drd2-Cre* line might contribute to these effects^19^, we used the same approach using *ChAT*-*Cre* mice. D2R overexpression selectively in cholinergic interneurons did not alter PR performance (Supplementary Fig. 1). These results indicate that D2R upregulation in indirect pathway projection neurons of the NAc core is sufficient to enhance the willingness to work for a reward.

We also evaluated the impact of D2R upregulation on effort-related decision-making using a concurrent choice task^3,18^. This paradigm measures the animals’ choice to work for a preferred reward (evaporated milk) when simultaneously presented with a less preferred but freely accessible reward (chow). When no chow was available (“no choice”), D2R-OE_NAcInd_ mice exhibited an overall increase in responding to the increasing ratio requirements compared to controls (*F*_(1,12)_ = 8.792, *p* < 0.05) (Fig. 1h). There was also an interaction of D2R upregulation with work requirement (*F*_(5,60)_ = 3.179, *p* < 0.05). This effect was particularly prominent at the highest ratio requirements RR80 and RR120 (Bonferroni post hoc test, *p* < 0.01), where D2R upregulation led to higher responding compared to EGFP. As expected, when chow was freely available in the operant chamber (“choice”), both groups showed a reduction in responding compared to the “no-choice” setting, especially at the more demanding ratio requirements (Fig. 1i). A 2-way ANOVA further indicates that, in the presence of chow, D2R-OE_NAcInd_ mice exhibited higher responding compared to EGFP_NAcInd_ mice (virus effect: *F*_(1,12)_ = 8.851, *p* < 0.05; virus × ratio interaction: (*F*_(5,60)_ = 3.302, *p* < 0.05). EGFP_NAcInd_ mice reduced their responding sooner than the D2R-OE_NAcInd_ mice and the performance difference between the two groups was measurable at a lower ratio when free food was available (RR40, Bonferroni post hoc test: *p* < 0.05) compared to RR80 in the “no-choice” setting. Together with the PR data, these results suggest that increased D2R levels in accumbal indirect pathway MSNs promote incentive motivation.

### D2R upregulation in D2-MSNs reduces inhibition of D1-MSNs

**Fig. 2:**
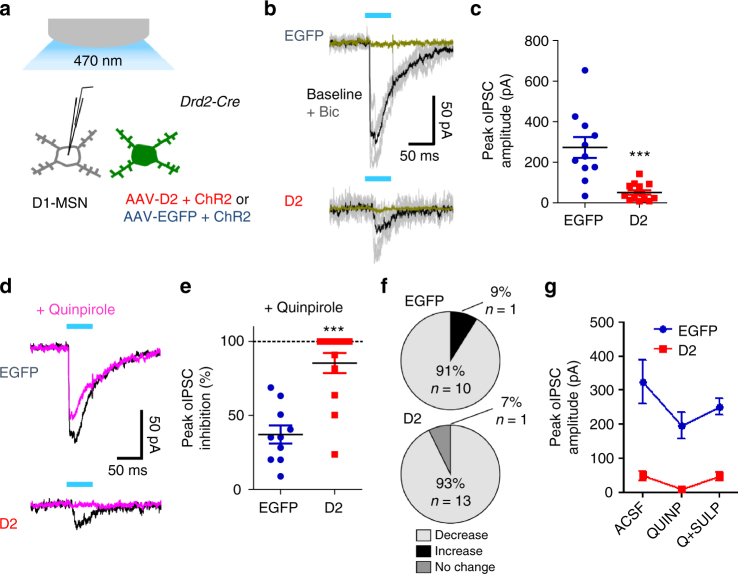
D2R upregulation in D2-MSNs leads to reduced local collateral transmission. **a** Inhibitory postsynaptic currents (IPSCs) were evoked by blue light illumination of MSNs co-expressing ChR2 and D2R or ChR2 and EGFP in NAc slices. oIPSCs were recorded from neighboring non-fluorescent MSNs. **b** Individual (gray) and average (black) traces oIPSC traces (gray) from representative MSNs following five 50-ms light pulses (1 Hz). Bicuculline (10 µM) eliminated the oIPSC. **c** Peak oIPSC amplitude under basal conditions was significantly reduced when D2Rs were overexpressed in D2-MSNs [*n* = 11 cells (6 mice) or 14 cells (9 mice)]. **d**, **e** Treatment with quinpirole (1 µM) reduced oIPSC amplitude in both groups, though to a greater extent in D2R-OE mice [*n* = 10 cells (6 mice) or 14 cells (9 mice)]. **f** Quinpirole showed an inhibitory effect on oIPSC amplitude in >90% of cells recorded in both groups. **g** Sulpiride co-treatment (1 µM) reversed the effect of quinpirole on peak oIPSC amplitude [*n* = 7 cells (4 mice) or 11 cells (8 mice)]. Error bars = s.e.m.

To identify neuronal mechanisms by which D2R upregulation enhances motivation we turned to whole cell patch clamp recordings in NAc slices. Due to the proposed role of presynaptic D2Rs in regulating transmitter release, we examined whether D2R upregulation affects the functional output of D2-MSNs. D2-MSNs extend local inhibitory axon collaterals to D1-MSNs of the direct pathway^38,40–44^. To determine the impact of D2R upregulation on D2-to-D1-MSN collateral transmission, we co-expressed channelrhodopsin-2 (ChR2) and D2R or EGFP in D2-MSNs. About 84% of ChR2 positive neurons co-expressed D2R-IRES-Venus, and we confirmed that the ability to induce action potentials by ChR2 activation in D2-MSNs was similar between groups (Supplementary Figs. 2 and 3). Optically-evoked inhibitory postsynaptic currents (oIPSCs) were recorded from the non-fluorescent, D1-MSN-enriched MSN population following photostimulation of D2-MSNs (Fig. 2a). In both groups of animals, we measured oIPSCs that were blocked by the GABA_A_ receptor antagonist bicuculline (10 µM) (Fig. 2b). Notably, we found that the peak amplitude of the oIPSCs was dramatically attenuated in D1-MSNs of D2R-OE_NAcInd_ mice compared to EGFP controls (51.1 ± 10.9 vs. 273.1 ± 51.6 pA; *t* = 4.709, *p* < 0.0001, *n* = EGFP: 11(6), D2: 14(9) cells/group) (Fig. 2b, c). Moreover, while the D2R agonist quinpirole (1 µM) attenuated the oIPSC in over 90% of recorded neurons in both EGFP_NAcInd_ and D2R-OE_NAcInd_ mice, the extent of quinpirole-mediated inhibition was much greater in the D2R-OE_NAcInd_ mice (85.4 ± 6.8 % vs. 37.1 ± 6.1 % in EGFP controls; *t* = 5.105, *p* < 0.0001) (Fig. 2d–f). When the D2R antagonist sulpiride (1 µM) was co-applied with quinpirole in a subset of cells that had been treated with quinpirole alone, the oIPSC amplitude was reversed to near basal levels in both groups (Fig. 2g). Together, these results suggest that D2R upregulation in D2-MSNs reduces collateral inhibition of D1-MSNs.

The enhanced quinpirole-mediated effect on oIPSC size observed in D2R-OE_NAcInd_ mice is consistent with enhanced presynaptic inhibition of GABA release mediated by activation of a greater number of presynaptic D2Rs. However, it remains unclear whether pre- or postsynaptic mechanisms are responsible for the basal reduction in oIPSC amplitude in D1-MSNs of these mice. D1-MSN intrinsic membrane properties and excitability were not altered in D2R-OE_NAcInd_ mice (Supplementary Fig. 4). In addition, analysis of miniature inhibitory postsynaptic currents (mIPSCs) in D1-MSNs revealed no significant effects of D2R upregulation on either mIPSC frequency or amplitude (Supplementary Fig. 5). However, in the slice preparation, inhibitory events onto MSNs are derived, to a great extent, from striatal interneurons^45^, so these measurements may not be sufficiently sensitive to detect changes in the collateral inputs from indirect pathway neurons.

Therefore, to achieve greater input-specificity, we replaced extracellular Ca^2+^ with strontium (Sr^2+^) and recorded from D1-MSNs following optogenetic stimulation of D2-MSNs (Supplementary Fig. 6). Under these conditions, Sr^2+^ leads to desynchronization of neurotransmitter release in terminals recently invaded by an action potential, generating asynchronous IPSCs (asIPSCs) that can be measured for several hundred milliseconds after the initial stimulus^46^. This technique has been used to directly assess postsynaptic efficacy (asIPSC amplitude) and, indirectly, presynaptic function or number of synaptic contacts (asIPSC frequency) in specific basal ganglia circuits^46–48^. We found that in this paradigm, D2R upregulation resulted in significantly decreased mean asIPSC amplitude and frequency even after normalization to the amplitude of the oIPSC (Supplementary Fig. 6). Together, these data suggest that the basal decrease in inhibitory transmission observed in D2R-OE_NacInd_ mice is due both to pre- and postsynaptic alterations, without associated changes in excitability or global inhibitory input.

### In vivo D1-MSN activity is not altered by D2R upregulation

**Fig. 3:**
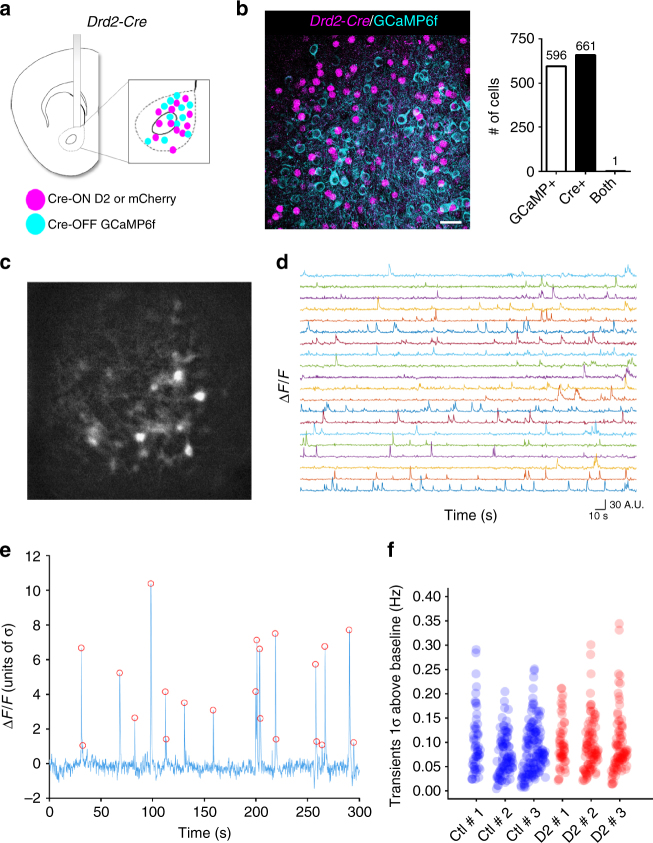
D2R upregulation in D2-MSNs does not alter D1-MSN activity in vivo. **a** Schematic of GRIN lens lowered into the NAc core of *Drd2-Cre* mice transduced with a Cre-ON D2-IRES-mCherry or mCherry in Cre (+) cells (primarily expressed in D2-MSNs) and a Cre-OFF GCaMP6f in Cre (–) cells (primarily expressed in D1-MSNs). **b** Double immunofluorescence analysis against Cre and GFP shows that in a *Drd2-Cre* mouse, Cre-OFF GCaMP6f (cyan) expression is restricted to Cre (–) cells. Scale = 25 μm. **c** Representative fluorescent image from live D1-MSNs expressing Cre-OFF GCaMP6f. **d** Individual neural Ca^2+^ transient traces from D1-MSNs in one representative mouse during progressive ratio task. **e** Ca^2+^ transients were defined as events with peak amplitudes greater than 1 standard deviation (S.D.) from baseline activity for analysis. Red circles denote transients whose amplitudes are above 1 S.D. **f** Frequency of Ca^2+^ transients recorded during PR from individual cells in 3 mice per group

We hypothesized that reduced collateral inhibition onto D1-MSNs might enhance direct pathway activity and thereby contribute to the increase in motivation. To this end, we expressed either DIO-D2R-ires-mCherry or DIO-mCherry in NAc D2-MSNs in *Drd2-Cre* mice, while simultaneously expressing the genetically encoded Ca^2+^ sensor GCaMP6f in D1-MSNs using a Cre-OFF FAS system^49^ (Fig. 3a). We verified that the FAS-GCaMP6f construct was targeted to non-Cre expressing cells in *Drd2-Cre* mice (Fig. 3b). A microendoscopic lens was implanted above the viral injection site to image putative D1-MSN Ca^2+^ activity during the PR task (Fig. 3c).

We recorded Ca^2+^ activity in a 5-min period in the PR test that encompasses the ratios with the highest press rates in both groups. First, we determined whether D2R upregulation was associated with changes in basal Ca^2+^ activity in D1-MSNs. We used the constrained non-negative matrix factorization framework modified for microendoscopes (CNMF-E) to localize neurons and extract denoised and demixed Ca^2+^ signals in our recordings^50^. We reliably detected low frequency, high amplitude Ca^2+^ transients, as reported in other studies of Ca^2+^ activity in MSNs^51^ (Fig. 3c–e). D2R upregulation, however, did not result in significant changes in the average frequency of Ca^2+^ transients (mCherry: 0.069 ± 0.006 Hz; D2: 0.082 ± 0.002 Hz; *t* = 1.97, *p* = 0.12, *n* = 3 mice/group) (Fig. 3f). Similar results were obtained when the threshold for transient detection was increased to 2 or 3 S.D. above baseline activity (2 S.D.: mCherry: 0.032 ± 0.002 Hz; D2: 0.035 ± 0.001 Hz; *t* = 1.73, *p* = 0.16; 3 S.D.: mCherry: 0.021 ± 0.001 Hz; D2: 0.021 ± 0.001 Hz; *t* = 0.383, *p* = 0.72; *n* = 3 mice/group). In addition, we sought to determine whether changes in Ca^2+^ signals were temporally associated with specific behavioral events during PR, such as lever pressing or reward approach. While we observed no clear relationship between Ca^2+^ activity and lever pressing (not shown), our analyses revealed that head entries into the reward port were associated with changes in Ca^2+^ activity, but only in a small number of D1-MSNs (Supplementary Fig. 7). Because these event-related changes in Ca^2+^ signals were not consistently observed across all rewards retrieved by one mouse and because they showed large variability within groups, no quantitative comparisons were drawn. Similar results were obtained when mice were run on a different food incentive operant task (FR5) (mCherry: 0.098 ± 0.007 Hz; D2: 0.118 ± 0.011 Hz; *t* = 1.405, *p* = 0.203, *n* = 4–5 mice/group) (Supplementary Fig. 8). These in vivo data suggest that D2R upregulation in D2-MSNs does not result in increased D1-MSN activity during the periods of highest responding in PR or in FR5.

### Accumbens D2Rs reduce inhibition of ventral pallidal neurons

**Fig. 4:**
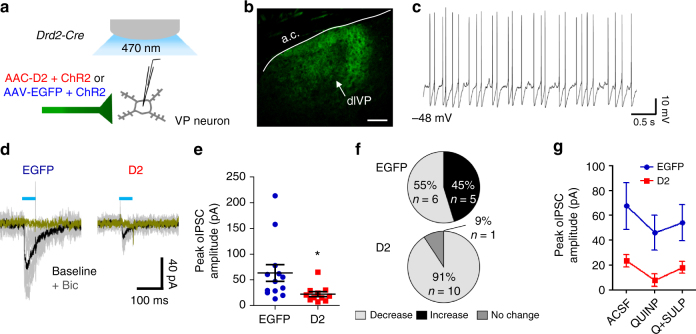
D2R upregulation reduces D2-MSN inhibitory transmission to ventral pallidum (VP). **a** Schematic of oIPSCs recordings from VP neurons in slice following blue-light stimulation of terminal fields from NAc MSNs co-expressing ChR2 and D2R or ChR2 and EGFP. **b** VP neurons were recorded from within virus-positive NAc D2-MSN terminal fields in the dorsolateral VP (dlVP). Scale bar = 150 µm. **c** Target neurons in VP typically displayed spontaneous firing activity, depolarized RMP, slow ramp-like depolarization preceding short duration spikes, and prominent afterhyperpolarization. **d** Individual (gray) and average (black) oIPSC traces (gray) from representative MSNs following five 50-ms light pulses (1 Hz). Bicuculline (10 µM) eliminated the oIPSC. **e** Peak oIPSC amplitude under basal conditions was significantly reduced when D2Rs were overexpressed in D2-MSNs MSNs [*n* = 13 cells (10 mice) or 11 cells (7 mice)]. **f** Quinpirole (1 µM) bidirectionally modulated oIPSCs recorded in EGFP_NAcInd_ VP neurons MSNs [*n* = 11 cells (9 mice) or 10 cells (6 mice)]. In contrast, quinpirole decreased the oIPSC amplitude in >90% of cells recorded in D2R-OE_NAcInd_ VP. **g** Opposite influences of D2 agonist and antagonist are highlighted by the overall effect of quinpirole and quinpirole + sulpiride (1 µM) on oIPSC amplitude. Error bars = s.e.m.

D2-MSNs in the NAc core send extensive projections to the ventral pallidum. We used an optogenetic approach similar to that used for the intra-NAc collaterals to test whether D2R upregulation in D2-MSNs reduces inhibitory transmission to the VP. We co-expressed ChR2 with either D2R or EGFP in NAc core D2-MSNs. Four weeks later, we recorded from VP neurons within dense, virus-positive terminal fields in slices of the dorsolateral VP (Fig. 4a, b), the primary target area of the NAc core^27^. This slice preparation did not include NAc (or D2-MSN cell bodies), allowing for selective light-evoked stimulation of D2-MSN terminals. Within the VP, we recorded oIPSCs from neurons with the characteristics described for putative GABAergic type B neurons in rat VP slices^52,53^ including spontaneous spiking, slow ramp-like depolarization preceding short duration spikes, and prominent afterhyperpolarization (Fig. 4c). The intrinsic properties of VP neurons recorded from both groups were comparable (resting membrane potential: EGFP, −46.5 ± 1.3 mV, *n* = 11 cells; D2, –49.8 ± 2.0, *n* = 13 cells; *t* = 1.3, *p* = 0.21; input resistance = EGFP, 317.1 ± 45.3 MΩ, *n* = 11 cells; D2, 590.7 ± 172.6, *n* = 13 cells; *t* = 1.4, *p* = 0.17). We found that D2R upregulation in D2-MSNs led to an approximately 65% reduction in oIPSC amplitude compared to controls after photostimulation of ChR2-positive D2-MSN terminals in the VP (Fig. 4d, e; *t* = 2.448, *p* < 0.05, *n* = EGFP: 13(10), D2: 11(7) cells/group). Under our stimulation conditions, we found that quinpirole (1 µM) had a bidirectional effect on oIPSC amplitude in control EGFP_NAcInd_ VP cells. After quinpirole, 55% of control VP neurons showed an attenuated response, whereas 45% showed an enhanced oIPSC (Fig. 4f and Supplementary Fig. 9). In contrast, quinpirole decreased oIPSC amplitude in >90% of VP cells in D2R-OE_NAcInd_ slices. The bias towards decreased oIPSCs in response to quinpirole in D2R-OE_NAcInd_ VP cells suggests that additional presynaptic D2Rs play an inhibitory role in regulating GABAergic transmission between NAc D2-MSNs and VP. Co-treatment with sulpiride (1 µM) reversed the oIPSC to near basal levels (Fig. 4g and Supplementary Fig. 9). Similar effects on oIPSC amplitude under basal conditions and following quinpirole were observed using light pulses of shorter duration (1 ms) (Supplementary Fig. 10).

### Inhibition of pallidal activity is reduced by D2Rs in vivo

**Fig. 5:**
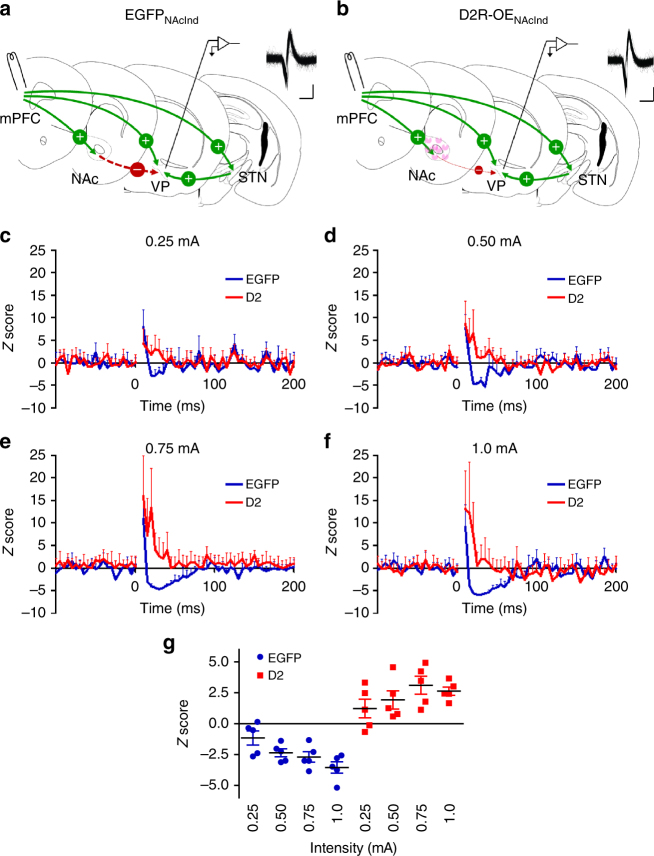
D2R upregulation is associated with reduced mPFC-evoked inhibition of ventral pallidal neurons in vivo. **a**, **b** Schematic depictions of the stimulating electrode in the medial prefrontal cortex (mPFC) and the recording electrode in the VP in anesthetized mice. mPFC stimulation can lead to excitation of VP neurons directly or via the subthalamic nucleus (STN). mPFC stimulation also leads to inhibitory responses in VP by activation of NAc GABAergic projection neurons. Solid green arrows and (+) sign depict excitatory projections, while the dotted red arrow and (–) sign represent inhibitory projections. Insets, representative VP spike waveforms from recorded cells (overlay of ~500 spikes; scale = 1.0 ms, 0.1 mV) are consistent with those of GABAergic neurons. We hypothesized that, in contrast to EGFP controls (**a**), additional D2Rs (pink) in NAc D2-MSNs would attenuate cortically-evoked inhibition of VP (**b**). **c**–**f** *Z* score transformed peristimulus time histograms (PSTHs) showing mPFC-evoked responses in all recorded VP neurons at different stimulation intensities. Electrical stimulation of the mPFC resulted in an excitation-inhibition response in the VP in the EGFP_NAcInd_ mice. VP neurons in D2R-OE_NAcInd_ mice generally lacked the inhibitory response. **g** Change in firing rate during the 75 ms following mPFC stimulation expressed as a *Z* score of the pre-stimulation firing rate distribution. Basal firing rates were not different between the two groups (EGFP_NAcInd_ mice: 8.83[1.67] Hz; D2R-OE_NAcInd_ mice: 11.18[2.08] Hz, *t*_22_ = −0.83, *p* = 0.42). EGFP_NAcInd_: *n* = 10 (5) units, D2R-OE_NAcInd_: *n* = 14 (5) units. Error bars = s.e.m.

We extended these findings from slice preparations by asking whether D2R upregulation also affects VP inhibition in an in vivo model in which cortico-basal ganglia loops remain intact. The medial prefrontal cortex (mPFC) exerts both excitatory and inhibitory regulation of VP neurons (Fig. 5a). Activation of the mPFC excites VP neurons directly through glutamatergic projections from the mPFC, as well as indirectly via the subthalamic nucleus^54–56^. It also leads indirectly to inhibition of VP neuron activity through its activation of NAc GABAergic projection neurons^55,57,58^. We hypothesized that, by decreasing D2-MSN output to VP, D2R upregulation in NAc D2-MSNs would decrease the inhibitory response of VP neurons to mPFC stimulation, while leaving the excitatory response unaffected (Fig. 5b). We performed single unit recordings in the VP of anesthetized D2R-OE_NAcInd_ and EGFP_NAcInd_ mice during electrical stimulation of mPFC (Fig. 5a, b). VP neuronal responses were assessed using peristimulus histograms (PSTHs) for 50 stimulus trials (0.2 ms pulse, 0.5 Hz) (Fig. 5c–f). To assess patterns in PSTHs, responses were first quantified using two-sample Kolmogorov–Smirnov (K–S) tests. While mPFC stimulation typically induced an excitation-inhibition response in VP neurons in EGFP_NAcInd_ mice, VP neurons in D2R-OE_NAcInd_ mice generally lacked the inhibitory response (K–S test: 0.25 mA: *Z* = 1.13, *p* = 0.15; 0.5 mA: *Z* = 1.70, *p* < 0.01; 0.75 mA: *Z* = 2.08, *p* < 0.01; 1.0 mA: *D* = 2.08, *p* < 0.01). VP neuronal responses were further quantified using *Z* score analysis, which revealed a significant current and genotype interaction (Fig. 5g) (EGFP_NAcInd_ mice: *F*_current [1,4]_ = 35.2, *p* < 0.01; D2R-OE_NAcInd_ mice: *F*_current [1,4]_ = 15.4, *p* = 0.02; *F*_genotype × current [1,8]_ = 44.34, *p* < 0.01). Thus, both ex vivo and in vivo results provide evidence for reduced indirect pathway-mediated inhibition of VP neurons as a consequence of D2R upregulation in accumbal D2-MSNs.

### Inhibiting accumbopallidal transmission enhances motivation

**Fig. 6:**
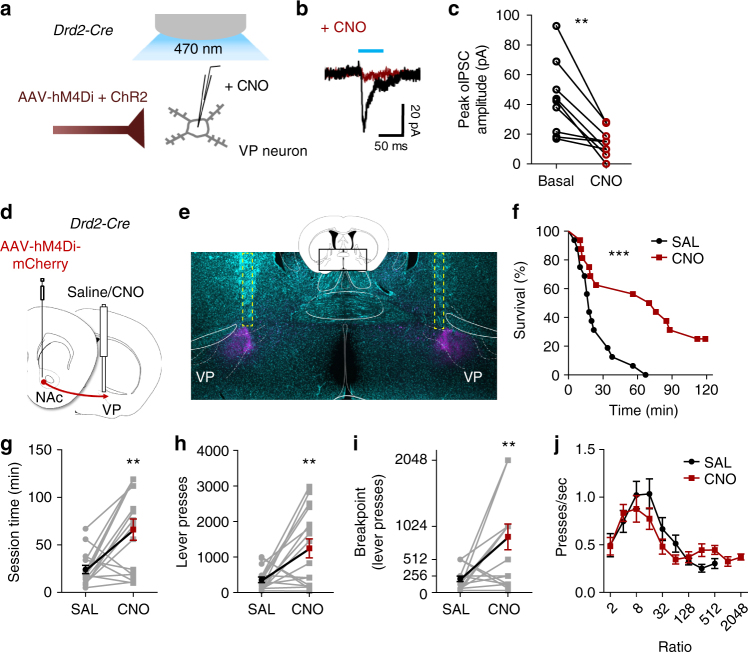
DREADD-mediated silencing of D2-MSN terminals in the VP enhances motivation. **a** Schematic illustrating oIPSC recording from VP neurons in slice following CNO (10 µM) treatment and blue-light stimulation of terminal fields from NAc MSNs co-expressing ChR2 and hM4Di. **b** Representative average oIPSC traces following 50-ms light stimulation under basal conditions (black) and after CNO application (dark red). **c** CNO significantly reduced oIPSC amplitude from baseline (14.5 ± 3.1 from 43.6 ± 8.3 pA; **p < 0.005, paired Student’s *t*-test, *n* = 9 (6) cells). **d** *Drd2-Cre* mice were injected hM4Di-mCherry AAV into the NAc, and a bilateral microinfusion cannula guide was implanted. Four weeks after surgery, an internal cannula was briefly inserted into the guide to deliver CNO (1 mM, 0.3 µl/side) or saline to the caudal VP prior to PR testing. All 16 mice received both treatments on different days in counterbalanced fashion. **e** Representative fluorescent Nissl stain (green) showing the location of bilateral microinfusion cannula tracks (yellow dotted lines) relative to hM4Di-mCherry immunofluorescent terminal fields (red) in the caudal aspect of the VP. Inset, coronal atlas plane (+0.02 mm relative to Bregma) from which image was acquired. **f**, **g** Significant enhancing effect of CNO microinfusion on session duration plotted as a survival function or as total session time (*n* = 16 mice/condition). **h**, **i** Lever presses and breakpoint were significantly increased after CNO (*n* = 16 mice/condition). **j** Press rate as a function of ratio requirement was not altered by CNO. Error bars = s.e.m.

Our slice electrophysiology results reported above are consistent with an inhibitory role of D2Rs located on D2-MSN terminals in VP. We therefore examined whether inhibition of D2-MSN synaptic transmission to the VP represents the neuronal mechanism by which D2R upregulation enhances motivation. We first co-expressed the DREADD hM4Di—which like D2R is G_i/o/z_-coupled—and ChR2 in NAc D2-MSNs. We then recorded oIPSCs in VP slices containing virus-positive D2-MSN terminals in the presence or absence of CNO (Fig. 6a). Similar to the inhibitory effects of quinpirole in D2R-OE_NAcInd_ mice, light-evoked IPSCs were significantly diminished by CNO (10 µM) in all VP neurons recorded (*t* = 4.284, *p* < 0.005) (Fig. 6b, c), as observed by Bock et al^31^. This suggests that G_i/o/z_-activation in D2-MSN terminals reduces GABAergic output onto VP neurons.

We then tested whether G_i/o/z_-mediated inhibition of D2-MSN projections innervating the VP alters PR performance. To this end, we bilaterally expressed hM4Di-mCherry in D2-MSNs of the NAc and implanted microinfusion bilateral cannulae targeting the mCherry-positive terminal fields in the caudal aspect of the dorsolateral VP (Fig. 6d, e and Supplementary Fig. 11). All mice underwent 2 PR sessions, prior to which they received local, bilateral microinfusions of CNO or saline (0.3 µl/side) in a counterbalanced fashion. Survival function analysis revealed a significant CNO effect on the percentage of mice that continued to respond as session time elapsed (Log-rank test, *χ*^2^ = 7.244, *p* < 0.001) (Fig. 6f). CNO also led to a significant increase in total session duration compared to saline treatment (*t* = 3.54, *p* = 0.005, *n* = 16/group) (Fig. 6g). In addition, the number of presses and the breakpoint were significantly enhanced (presses: *t* = 3.054, *p* < 0.01; breakpoint: *t* = 3.128, *p* < 0.01, *n* = 16/group) (Fig. 6h, i), while press rate was unaltered (Fig. 6j). CNO injection did not enhance motivation in the absence of hM4D expression (Supplementary Fig. 12). These results indicate that inhibition of the D2-MSN projections just within the VP is sufficient to enhance motivation.

## Discussion

We have found that selective upregulation of D2Rs in the indirect pathway of the NAc increases motivated behavior. This is associated with decreased inhibitory transmission to two main D2-MSN targets, neurons in the ventral pallidum and MSNs of the direct pathway. In vivo, D2R upregulation was associated with decreased inhibition of the VP, but not of putative D1-MSNs. Moreover, inhibition of synaptic transmission from D2-MSNs to the VP is sufficient to enhance motivation. Our data therefore suggest that D2R upregulation promotes motivated behavior by weakening the canonical indirect pathway projections to the VP.

Earlier studies using lesions or localized infusions of GABA agonists or antagonists into the VP have shown the importance of this region in motivated behavior^59^. Moreover, local injections of the GABA agonist muscimol into the subthalamic nucleus (STN), one synapse downstream of the VP, enhanced PR performance^60^. Thus, we propose that disinhibition of the VP as achieved by D2R upregulation in NAc D2-MSNs or by increased G_i/o_ signaling via hM4Di activation in D2-MSN terminals, may invigorate PR performance, in part, through a reduction of STN function.

Our results also implicate altered local collateral inhibition of the direct pathway as a mechanism by which D2R upregulation enhances motivation. Studies using paired recordings or optogenetic tools have shown significant functional connectivity between MSNs in the striatum^38,40–44,61^. Generally, D2-MSNs have a stronger influence on D1-MSNs than D1-MSNs on D2-MSNs^43^. Here, we observed collateral inhibition from D2-MSNs to virus-negative MSNs, which in the NAc core are enriched in D1-MSNs of the direct pathway. This collateral inhibition was strongly reduced in D2R-OE_NAcInd_ mice.

However, despite these findings in the slice preparation, using in vivo Ca^2+^ imaging during the PR task, we found no significant effect of D2R upregulation on D1-MSN activity. This suggests that a reduction in D2-MSN-to-D1-MSN transmission is not sufficient to alter overall activity of direct pathway neurons during tasks of incentive motivation, thus arguing against disinhibition of D1-MSNs as a principal mechanism underlying the increase in motivation seen following D2R upregulation. It is possible that the role of D2Rs in lateral inhibition between D2 and D1-MSNs, however, is more behaviorally relevant in settings of high dopamine release, as in the behavioral response to psychostimulants^61^.

D1-MSNs of the NAc also send projections to the VP in addition to their well-known projections to the midbrain^29^, analogous to striatopallidal D1 “bridging” collaterals in the dorsal striatum^22^. These projections might also play a role in regulating motivation. However, our in vivo analysis did not identify alterations in D1-MSN activity, suggesting that this pathway may not be affected in D2R-OE_NacInd_ mice.

Our slice physiology results show that D2R upregulation in D2-MSNs leads to a profound reduction in light-evoked inhibitory transmission to both local and distal targets via two mechanisms. First, we observed pre- and postsynaptic adaptations that reduce the basal strength of these connections. Acute sulpiride (D2R antagonist/inverse agonist) failed to restore the basal oIPSC amplitude in D2R-OE_NAcInd_ mice to control levels, arguing against ambient dopamine or constitutive activity of the D2Rs in the slice as a mediator of the inhibition of synaptic transmission. The strontium experiments revealed a reduction in both the frequency and amplitude of asIPSCs that was independent of the initial stimulus strength. These data, therefore, point to changes both at the level of presynaptic release (or number of synaptic contacts), and at the level of postsynaptic efficacy. Defining the precise mechanisms underlying these synaptic adaptations in basal D2-MSN output will be of interest for further investigation.

Second, acute treatment with the D2R agonist quinpirole attenuated light-evoked inhibitory currents in the NAc, an effect that was enhanced in D2R-OE_NacInd_ mice. This enhancement is likely due to decreased presynaptic function mediated by increased D2R levels, consistent with reports of acute presynaptic effects of quinpirole or dopamine on MSN-MSN or MSN-pallidum transmission^37–40,61^. Application of CNO to hM4Di-expressing D2-MSNs also resulted in acute attenuation of oIPSC in VP neurons, similar to the inhibitory effect of quinpirole in D2R-OE_NAcInd_ mice, suggesting that G_i/o/z_ coupled-signaling may be involved in both cases.

In our experiments, light-evoked IPSCs at D2-MSN to D1-MSN synapses were more sensitive to quinpirole-mediated inhibition than at striatopallidal synapses in EGFP_NAcInd_ mice, in agreement with other reports^61^. Over 90% of D2-to-D1-MSN oIPSCs were reduced by quinpirole whereas only 55% of striatopallidal oIPSCs transmission were reduced by the D2 agonist. Surprisingly, quinpirole increased the oIPSC in 45% of VP cells. The interpretation of the quinpirole effect is complicated by the fact that inhibition within the pallidum is also mediated by intra-pallidal connections of GABAergic neurons^62^, some of which also express D2/D3/D4 receptors^56,63–65^. Indeed, in vivo studies measuring VP firing rates after iontophoretic application of quinpirole found that about 24% of VP neurons increased their firing rates as expected but 15% of VP neurons decreased their firing rates^66^. This bidirectional effect may be due to D2-like receptors expressed within the VP. In D2R-OE_NAcInd_ mice, where D2Rs are selectively increased in D2-MSNs of the NAc, the balance was altered and the oIPSC were reduced in 90% of neurons in the presence of quinpirole. This suggests that presynaptic D2Rs at ventral striatopallidal terminals inhibit neurotransmission, as proposed in the GPe^37,39^. Besides showing enhanced quinpirole-mediated inhibition of oIPSC in VP slices containing no NAc neurons, D2R-OE_NAcInd_ mice displayed robust viral-mediated D2R immunofluorescence in D2-MSN terminal fields innervating the VP, further supporting a presynaptic role for D2Rs. In this respect, ultrastructural studies in VP have shown that most D2R-positive axon terminals are GABAergic^65^. Although a role for dopamine modulation of VP activity has been consistently reported, the role of presynaptic D2R function in the VP is less well understood, likely due to a lack of cell-type specific approaches^56^. Here, we provide new evidence linking D2Rs specifically found in afferent D2-MSN terminals to VP neuron activity.

A recent study reported that optogenetic inhibition of the NAc indirect pathway led to a decrease in progressive ratio performance^67^, whereas here decreasing indirect pathway function enhances motivation. However, there are important differences between both studies in the site of inhibition (unilateral versus bilateral inhibition), in the time window of inhibition (10 s vs. the entire session), and in the design of the task (inhibition of activity during a cue that signals the onset of the trial versus no explicit cue). It is therefore possible that the indirect pathway may have opposite functions regarding motivation depending on whether it is active during a cue signaling later reward or during the sustained process of working in the form of lever pressing. Moreover, unlike neuronal inhibition during a discrete time point in the task^67^, our approach is designed to turn up the gain for endogenously released dopamine acting on indirect pathway D2Rs.

Higher D2R levels in ventral striatum have been linked to higher trait motivation in ADHD and better treatment outcomes for drug abusers^10,68^. However, it has been unclear how altered D2R function affects ventral striatal circuitry and behavior. Here we show that selective D2R upregulation in indirect pathway MSNs is sufficient to invigorate motivated behavior by decreasing indirect pathway output. While our cell-selective D2R upregulation in the mouse is not intended to model a specific disorder, it has helped to provide mechanistic insight into the role of NAc D2Rs in motivation. This should have clinical relevance since D2R binding is altered in brain disorders with altered motivation^9–15^. Our results suggest that D2R-based or related strategies for decreasing ventral striatopallidal transmission may have the potential to advance therapies for reversing motivational deficits across multiple brain disorders. In particular, the adenosine A_2A_ receptor (A_2A_R) has been proposed as a promising therapeutic target for the motivational symptoms of depression^69^. A_2A_Rs are co-expressed with D2Rs in indirect pathway MSNs and are thought to oppose D2R function^70^. Indeed, A_2A_R antagonism within the NAc has been shown to enhance motivated behavior in rats^69,71^. Our results suggest that the mechanisms by which A_2A_R antagonists increase motivation may involve increased D2R function in D2-MSNs, and thus a weakening of indirect pathway output.

## Methods

### Mice

Adult male and female *Drd2-Cre* (ER44; GENSAT), *Drd2-Cre/D1-tdTomato* (*B6.Cg-Tg(Drd1a-tdTomato)6Calak/J*; Jackson); *ChAT*-*Cre* (GM60; GENSAT) backcrossed onto C57BL/6 J background were used in experiments. Mice were housed 3–5 per cage for most experiments or individually for Ca^2+^ imaging experiments, on a 12 h light/dark cycle. All experiments were conducted in the light cycle. All experimental procedures were conducted following NIH guidelines and were approved by Institutional Animal Care and Use Committees by Columbia University and New York State Psychiatric Institute.

### Surgical procedures

Mice (≥8 weeks old) were bilaterally injected with a previously characterized Cre-dependent double-inverted open reading frame (DIO) adenoassociated viruses (AAVs) encoding D2R-ires-Venus^19^, EGFP, ChR2-EYFP, hM3Dq-mCherry, or hM4Di-mCherry (UNC Vector Core, Chapel Hill, NC) into the nucleus accumbens (NAc) using stereotactic Bregma-based coordinates:^73^ AP, + 1.70 mm; ML, ± 1.20 mm; DV, –4.1 mm (from dura). Groups of mice used for experiments were first assigned their AAV-genotype in a counterbalanced fashion that accounted for sex, age, home cage origin. For cannula-guided microinfusion experiments, mice were implanted in caudal VP with a bilateral guide cannula (AP, + 0.12 mm; ML, ± 1.5 mm; DV, −2.5 mm (from dura) at the time of AAV surgery. On test days, following brief restraint of the mice, dummy cannulae were removed and internal cannulae (33 G) with a 2 mm projection were inserted into guide to achieve a final depth of –4.5 mm. The guide cannulae (26 G; Plastics One, Roanoke, VA) were anchored with machine microscrews and fixed in place with dental cement. Dummy cannulae were placed into the guide and protected with a dust cap. For Ca^2+^ imaging of NAc, a 0.5 mm craniotomy was made at the following coordinates: AP: + 1.7 mm, ML: + 1.2 mm (right side). Brain tissue was then carefully aspirated by lowering aspiration needle at a rate 0.1 mm/min until a depth of –2.4 mm (from skull surface). After aspiration, a 1:1 mix of AAV-DIO-mCherry (Addgene) or AAV-DIO-D2-IRES-mCherry AAV and a Cre-OFF AAV-FAS-GCaMP6f (constructed in-house using pAAV-Ef1a-FAS-hChR2(H134R)-mCherry-WPRE-pA from Bernardo Sabatini and produced at Vector Biolabs) was bilaterally injected (AP: + 1.7 mm, ML: ± 1.2 mm, DV: −4.2, −4.1, and −4.0 mm; 0.22 µl at each depth). A 0.5 mm diameter gradient index (GRIN) lens affixed to an imaging cannula (Doric Lenses, Inc.) was then carefully lowered through same skull opening to D/V: −4.1 mm. Lens cannula was anchored with machine microscrews and fixed in place with dental cement. Mice were imaged 5–6 weeks after cannula implantation.

### Operant apparatus

Eight operant chambers (model Env-307w; Med-Associates, St. Albans, VT) equipped with liquid dippers were used. Each chamber was located in a light- and sound-attenuating cabinet equipped with an exhaust fan, which provided 72-dB background white noise in the chamber. The dimensions of the experimental chamber interior were 22 × 18 × 13 cm, with flooring consisting of metal rods placed 0.87 cm apart. A feeder trough was centered on one wall of the chamber. An infrared photocell detector was used to record head entries into the trough. Raising of the dipper inside the trough delivered a drop of evaporated milk reward. A retractable lever was mounted on the same wall as the feeder trough, 5 cm away. A house light located on wall opposite to trough illuminated the chamber throughout all sessions.

### Dipper and lever press training

Four weeks after AAV surgery, mice underwent operant training. Mice were weighed daily and food-restricted to 85–90% of baseline weight; water was available ad libitum. In the first training session, 20 dipper presentations were separated by a variable inter-trial interval (ITI) and ended after 20 rewards were earned or after 30 min had elapsed, whichever occurred first. Criterion consisted of the mouse making head entries during 20 dipper presentations in one session. In the second training session, criterion was achieved when mice made head entries during 30 of 30 dipper presentations. For lever press training, lever presses were reinforced on a continuous reinforcement (CRF) schedule. Levers were retracted after each reinforcer and were presented again after a variable ITI (average 30 s). The reward consisted of raising the dipper for 5 s. The session ended when the mouse earned 60 reinforcements, or one hour elapsed, whichever occurred first. Sessions were repeated daily until mice achieved 60 reinforcements.

### Fixed interval (FI) training

In FI training, lever presses were reinforced until after a fixed interval (timed relative to the lever extension) had elapsed. Each reinforcement was followed by a variable inter-trial interval (average 30 s) during which the lever remained retracted, and then a new trial started, signaled by lever extension. Mice began with FI-4 s session and proceeded successively to longer interval sessions after earning ≥ 30 rewards in each session. The FI durations were 4, 8, 12, 16, and 24 s.

### Progressive ratio (PR) and fixed ratio (FR) tasks

Mice underwent operant training 4 weeks after surgery followed by PR. In PR, a reward was obtained after the mice made the required number of lever presses. The criterion was set at two lever presses for the first trial and the requirement doubled with each successive trial. The session ended after 2 h or after 3 min had elapsed without a lever press. Breakpoint was defined as the last criterion successfully completed. Mean values from 3 PR sessions were analyzed. For hM4Di experiments, CNO (0.3 µl, 1 mM in saline) or saline was delivered bilaterally to VP via cannula-based microinfusions using a Hamilton syringe and a microdrive motor at a rate of 0.05 µl/min. At the end of each infusion, the internal cannula was left in place for 15 min to ensure diffusion, and mice were tested on PR task 5 min after removing the internal and replacing the dummy. Each mouse received one infusion of saline and one of CNO, 3 days apart. Investigators were blind to genotype of mice during the experiment, and the order of the infusions was counterbalanced between groups, matched for age and sex. Mice had two intervening RR5 sessions in the days between each PR test.

### Concurrent choice task

For this task, we used random ratio (RR) schedules, which involve a constant probability of reinforcement for each lever press and assess effort in instrumental responding^18^. The mice were first trained for 3 days in 30 min RR5 sessions. Animals were then tested in concurrent RR/choice procedure. This task consisted of having 8–12 g of lab chow freely available in a dish in the operant chamber while the mouse performed the RR schedule. Increasing ratios with and without chow were used (RR5, RR10, RR20, RR40, RR80, RR120). One animal was excluded from the data analysis because of lever pressing extinction.

### Slice preparation and whole-cell patch clamp recording

Four weeks after surgery, brains were harvested into ice-cold, oxygenated ACSF containing (in mM): 1.25 NaH_2_PO_4_, 2.5 KCl, 10 glucose, 26.2 NaHCO_3_, 126 NaCl, 2 CaCl_2_ and 2 MgCl_2_ (pH 7.4, 300–310 mOsm). Coronal striatal slices (300 μm) were cut on a vibratome in ice-cold, oxygenated ACSF and incubated at 32 °C for 30 min followed by 1 h at room temperature before recording. Voltage- and current-clamp whole-cell recordings were performed using standard techniques at room temperature. Electrodes were pulled from 1.5 mm borosilicate-glass pipettes on a P-97 puller (Sutter Instruments). Electrode resistance was ~4–6 MΩ when filled with internal solution consisting of (in mM): 130 K^+^-Gluconate, 5 NaCl, 10 HEPES, 0.5 EGTA, 2 Mg^+^-ATP, and 0.3 Na^+^-GTP (pH 7.3, 280 mOsm). Whole-cell patch-clamp recordings were obtained with a Multiclamp 700B amplifier, digitized at 10 kHz using a Digidata 1440 A acquisition system with Clampex 10, and analyzed with pClamp 10 (Molecular Devices). Only cells that maintained a stable access resistance (<30 MΩ) throughout the entire recording were analyzed. GFP/Venus or tdTomato positive MSNs within the NAc core were identified under IR-DIC optics and epifluorescence microscopy. Current–voltage and input–output (spike frequency) curves were obtained from injecting 500 ms currents ranging from −150 to +340 pA in 10 pA steps. Spiking frequency (Hz) was determined from the initial pair of action potentials^72^. mIPSCs were isolated by adding TTX (1 µM), D-AP5 (50 µM), and CNQX (20 µM) in ACSF while recording at a holding potential of −80 mV and measured over a period of 6 min using Synaptosoft Mini Analysis software. To enhance detection of mIPSCs, we used high Cl^−^ internal solution, consisting of (in mM): 136.5 KCl, 10 HEPES, 0.5 EGTA, 2 Mg^+^-ATP, and 0.3 Na^+^-GTP (pH 7.3, 280 mOsm). Light-evoked IPSC experiments were done at RT at –80mV in the presence of D-AP5 (50 µM) and CNQX (20 µM). oIPSCs were evoked with five 1-ms or 50-ms light pulses (470 nm, 1 Hz, 2.3 mW) using field illumination through a ×40 objective with a PE-100 CoolLED illumination system (Olympus). High Cl^−^ internal solution, as shown above, was used to enhance detection of oIPSCs. We blocked oIPSCs using 10 µM bicuculline. Peak amplitudes of oIPSCs were measured from averages of five individual traces. Slices were treated for 5 min with quinpirole hydrochloride (1 µM), sulpiride (1 µM) or clozapine N-oxide (10 µM). To measure asynchronous IPSCs, recordings were conducted at 32 °C in extracellular solution containing 2 mM Sr^2+^ (0 mM Ca^2+^), D-AP5 (50 µM) and CNQX (20 µM), while holding at –70 mV. Internal solution was as for mIPSC solution with substitution of KCl with 136.5 mM CsCl and the addition of 2 mM QX-314 hydrobromide. Ten trials using a 5-ms blue light pulse (0.6 Hz) were used to activate ChR2-positive terminals and generate asIPSCs. Detection of asIPSCs occurred within the 50–500 ms period after the stimulus^47^. asIPSCs were detected if greater than 2 S.D. above noise using Synaptosoft MiniAnalysis software, and the amplitude of each event was measured from a local baseline 5 ms before the start of peak to avoid decaying transient artifacts associated with residual synchronous release^46^.

### In vivo single unit recordings

Mice anesthetized with chloral hydrate were implanted with a stimulating electrode (Tungsten concentric, Microprobes) for electrical stimulation of the mPFC (AP + 1.9, ML ± 0.3, DV −2.0). A glass electrode (impedance 12–14 MΩ) filled with 2 M NaCl was lowered into the VP (AP −0.1, ML ± 1.5, DV −4.0) using a hydraulic microdrive to detect spontaneously active pallidal neurons. From this starting point, the VP was sampled in four locations 0.15 mm apart and arranged in a 2 × 2 spaced grid. The starting locations were counterbalanced across animals and groups. Pallidal neurons were identified using a combination of stereotaxic position and narrow action potential width (<1 ms). After 2–3 min of stable recording, electrical stimulation (50 pulses, 0.2 ms pulse width, 0.5 Hz) was applied at 0.25, 0.5, 0.75, and 1.0 mA current intensities. Neuronal activity was amplified and filtered (1000 × gain, 100–10 KHz band pass) and fed to an audio monitor and to a computer interface with custom-designed acquisition and analysis software (Neuroscope). Peri-stimulus time histograms (PSTHs) were obtained by sampling spike frequency with 5 ms bins 200 ms before, during and 200 ms after electrical stimulation. Spike frequencies in the PSTHs were expressed as *Z* scores expressed as a function of baseline firing during the 200 ms preceding electrical stimulation. Average firing rate over the first 75 ms following electrical stimulation was then compared between the 2 groups.

### In vivo calcium imaging

For Ca^2+^ imaging experiments, mice were habituated to a dummy microscope and cables during final phases of FI training. Prior to PR test, a Doric Lenses snap-in microscope was attached to the imaging cannula under brief isoflurane anesthesia. Ten minutes after full recovery, mice were placed in operant chamber to begin PR session. Image acquisition was done at 10 Hz, triggered by presentation of the 3rd reward (completion of ratio 8) and lasting for 5 consecutive minutes, and was conducted over 2 daily PR sessions. This period was chosen because it generally showed the highest press rates, generated more than 1 reward, and showed no difference in responding between groups. FR5 sessions, in which mice must make 5 presses to earn a reward, were conducted similarly over 5 min, but with image acquisition starting at session onset.

Image stacks collected using the microendoscope imaging system were registered using Doric Neuroscience Studio Image Analysis software. Each stack was then imported into the CNMF-E framework as previously described^50^. CNMF-E was used to locate neurons and extract raw Ca^2+^ fluorescence values over time. Neural Ca^2+^ transients were defined as events whose Δ*F*/*F* peak amplitudes were greater than 1 standard deviation above the baseline fluorescence. Custom MATLAB functions and scripts were used to import and analyze MedPC (MedAssociates) behavioral data corresponding to each of the fluorescence traces.

### Histology

Mice were transcardially perfused with ice-cold 4% paraformaldehyde (Sigma, St. Louis, MO) in PBS under deep anesthesia. Brains were harvested, post-fixed overnight and washed in PBS. Free-floating 30-µm coronal sections were obtained using a Leica VT2000 vibratome (Richmond, VA). After incubation in blocking solution (10% fetal bovine serum, 0.5% bovine serum albumin in 0.5% TBS-Triton X-100) for 1 h at room temperature, sections were labeled overnight at 4 °C with primary antibodies against GFP/mVenus/GCaMP6f (chicken; 1:1000; AB13970 Abcam, Cambridge, MA); D2R (rabbit; 1:500; in-house); Cre (rabbit, 1:2000; in-house^74^); and DsRed (rabbit; 1:250; 632496, Clontech, Mountain View, CA) to label mCherry. Sections were incubated with fluorescent secondary antibodies for 1 h at RT followed by, in some cases, incubation in Neurotrace^®^ green fluorescent Nissl stain according to manufacturer’s protocol (Life Technologies, Grand Island, NY). Sections were then mounted on slides and coverslipped with Vectashield containing DAPI (Vector, Burlingame, CA). Digital images were acquired using a Nikon epifluorescence microscope, or with a Leica SP8 scanning confocal microscope, and processed with NIH Image J and Adobe Photoshop software.

### Data analysis

Sample sizes were determined by performing statistical power analyses based on effect sizes observed in preliminary data or on similar work in the literature. Statistical analyses were performed using Graphpad Prism 5.01. Data are expressed as mean ± SEM. Paired and unpaired two-tailed Student’s *t*-tests were used to compare 2-group data, as appropriate. Multiple comparisons were evaluated by one-way or two-way ANOVA and Bonferroni’s post hoc test, when appropriate. Log-rank tests were used to analyze survival curves. A *p*-value of <0.05 was considered statistically significant. Behavioral and electrophysiological findings were successfully replicated with mice from different litters, ages, or sexes, and in several instances, across independent cohorts or related mouse strains. In addition, evoked electrophysiology responses were replicated using different stimulus intensities in vitro and in vivo, and data was collected from several animals.

### Code availability

Custom code used to generate results that are reported in the paper will be made available upon reasonable request.

### Data availability

The data that support the findings of this study are available from the corresponding author upon reasonable request.

## Electronic supplementary material

Supplementary Information
