# Supplementary material for: Accumbens dopamine D2 receptors increase motivation by decreasing inhibitory transmission to the ventral pallidum
Source: Nat Commun. 2018 Mar 14;9:1086. doi: 10.1038/s41467-018-03272-2 (PMC5852096; doi:10.1038/s41467-018-03272-2)
Supplement: Supplementary file 1 — Supplementary Information [file 41467_2018_3272_MOESM1_ESM.pdf]

ChAT-Cre

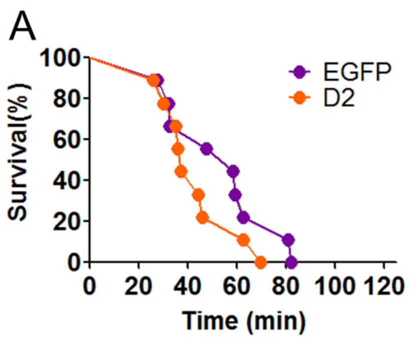

Supplementary Figure 1. D2R upregulation in NAc cholinergic interneurons does not alter progressive ratio performance. ***A.*** Kaplan-Meier survival function showing no significant alterations in PR responding over time between EGFP or D2-overexpressing *ChAT*-Cre mice (n = 8 or 9 mice/group). ***B, C***. D2R upregulation in cholinergic interneurons did not affect lever pressing or breakpoint (n = 8 or 9 mice/group). Error bars = s.e.m.

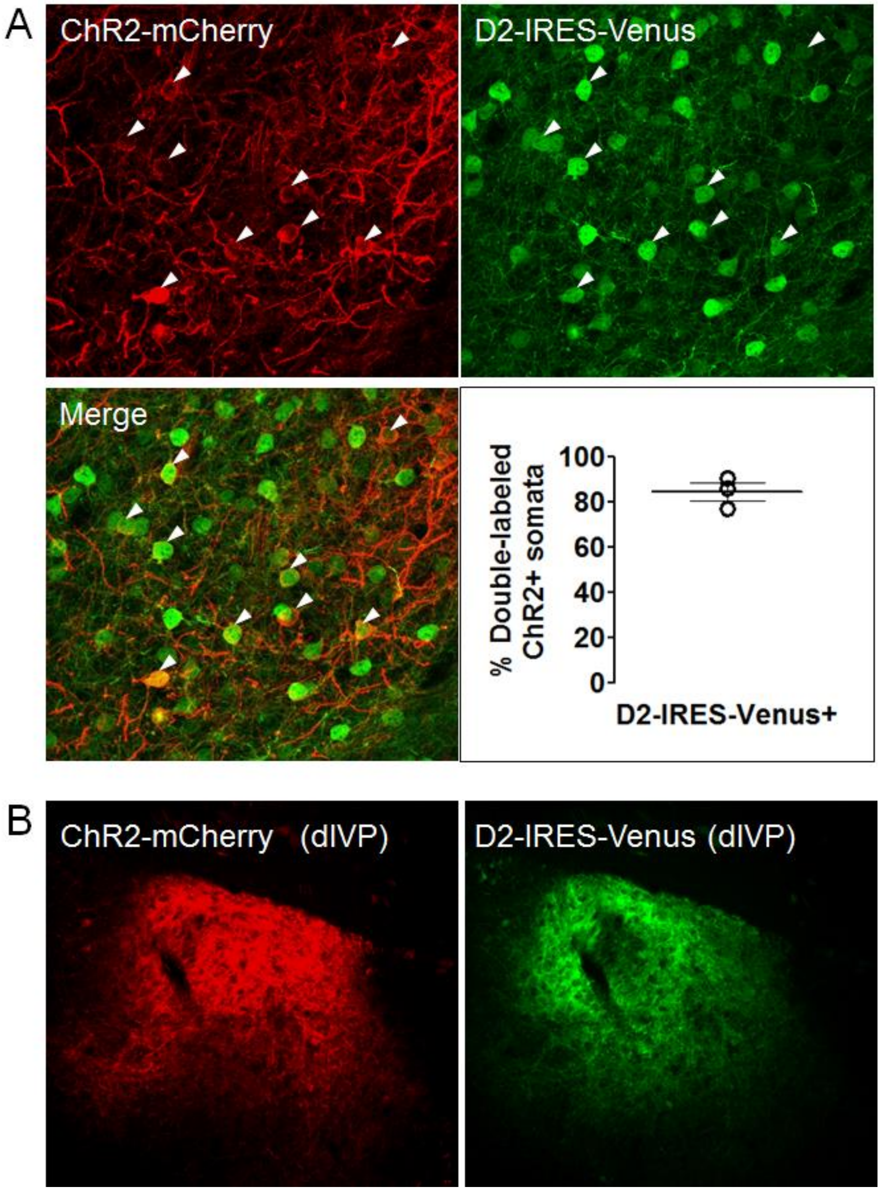

Supplementary Figure 2. ChR2-positive cells express D2R AAV. ***A.*** Top panel, confocal immunofluorescence imaging of ChR2-mCherry+ cells (red) and D2-IRES-Venus+ cells (green) in the NAc. Lower panel (*left*), merged channels showing overlap of ChR2+ and Venus+ cells. Given the dense neuropil-like immunolabeling of ChR2 fusion proteins like ChR2-mCherry, we relied on the relatively few ChR2-mCherry+ somata for analysis of co-localization with the somatic labeling of D2-IRES-Venus. Colocalization was therefore expressed as % of ChR2+ somata that is also Venus+. *Right*, mean co-expression from 3 mice. Co-expression was observed in 421 of 500 (84.2%) ChR2+ cells. ***B.*** Examination of the dorsolateral VP revealed substantial overlap of ChR2+ (red) and Venus+ (green) D2-MSN terminal fields. Error bars = s.e.m.

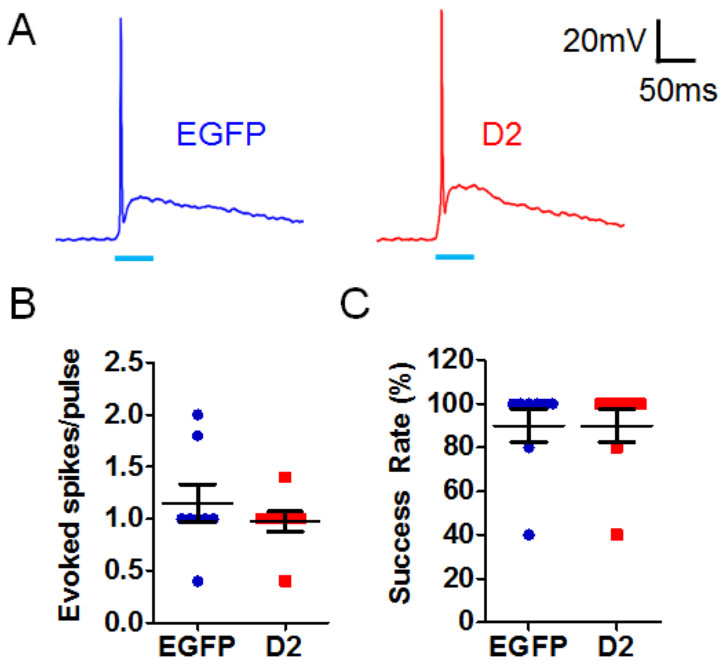

Supplementary Figure 3. **ChR2 response in D2-MSNs is not affected by D2R upregulation**. *A.* Current clamp recordings show a similar ChR2 response to a 50-ms light pulse in ChR2/EGFP and Chr2/D2R co-expressing D2-MSNs following a 50-ms blue-light pulse. *B.* The number of spikes evoked by each of 5 light pulses was averaged. No significant difference between groups was found. *C.* The success rate, defined as the percentage of pulses eliciting at least one spike, was not significantly altered by D2R upregulation [n = 8 cells (7 mice) or 8 cells (8 mice)]. Error bars = s.e.m.

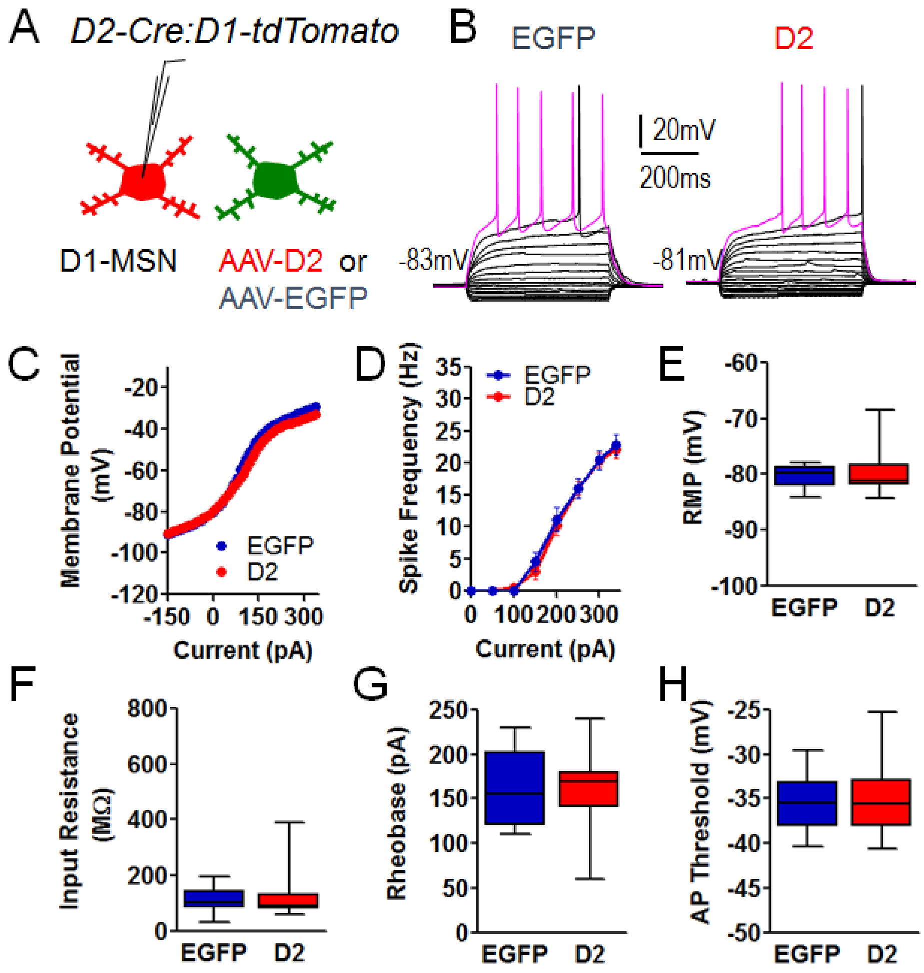

Supplementary Figure 4. D2R upregulation in D2-MSNs does not alter D1-MSN excitability. ***A.*** Schematic illustrating whole-cell recordings from tdTomato-positive, D1-containing MSNs neighboring MSNs that express either D2 or EGFP in the NAc of D2-Cre/D1-tdTomato mice. ***B.*** Representative current clamp recordings from tdTomato-expressing MSNs in *D2*-Cre x *D1*-tdTomato mice (500 ms current pulses from -140 pA to rheobase in 20 pA steps). Fuchsia traces showing the firing elicited at +230 pA reveal no effect of D2R upregulation in D2-MSNs on D1-MSN evoked firing. Neither current–voltage (***C***) nor input–output (***D)*** curves were significantly altered in D1-MSNs. Box plots showing that resting membrane potential **(*E*)**, input resistance **(*F*)**, rheobase **(*G*)**, or action potential threshold **(*H*)** were not significantly altered in D1-MSNs of D2R-OE_{NAcInd} mice [n = 14 cells (6 mice) or 15 cells (5 mice)]. Boxplots show median, lower and upper quartiles (box), and minimum and maximum (whiskers).

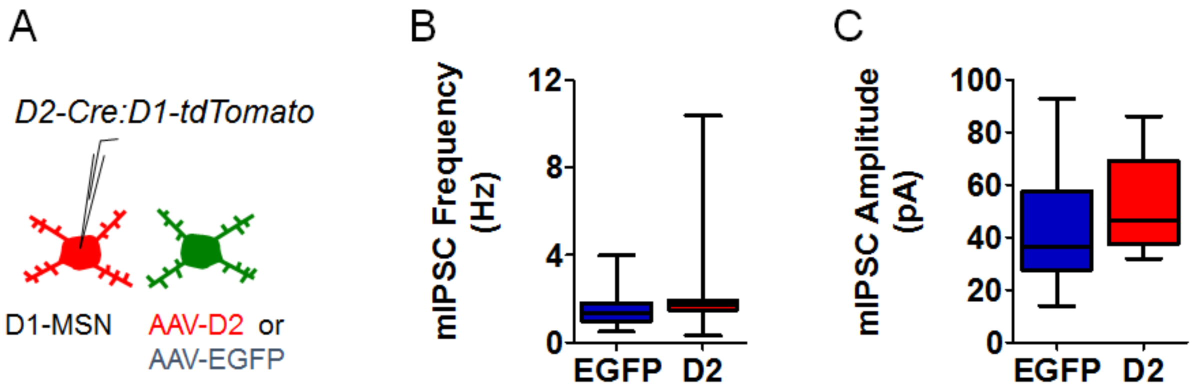

Supplementary Figure 5. D2R upregulation in D2-MSNs does not alter inhibitory input to D1-MSNs. ***A.*** Whole-cell recordings in the presence of 1 μM TTX were performed to isolate miniature inhibitory postsynaptic currents (mIPSCs) from tdTomato-positive, D1-containing MSNs, following EGFP or D2 overexpression in D2-MSNs. ***B.*** No significant alterations in frequency were observed (t = 1.237, p = 0.23) ***C.*** Amplitude of mIPSCs was not significantly affected [*t* = 1.342, p = 0.19; n = 14 (5) or 16 (4) cells/group]. Boxplots show median, lower and upper quartiles (box), and minimum and maximum (whiskers).

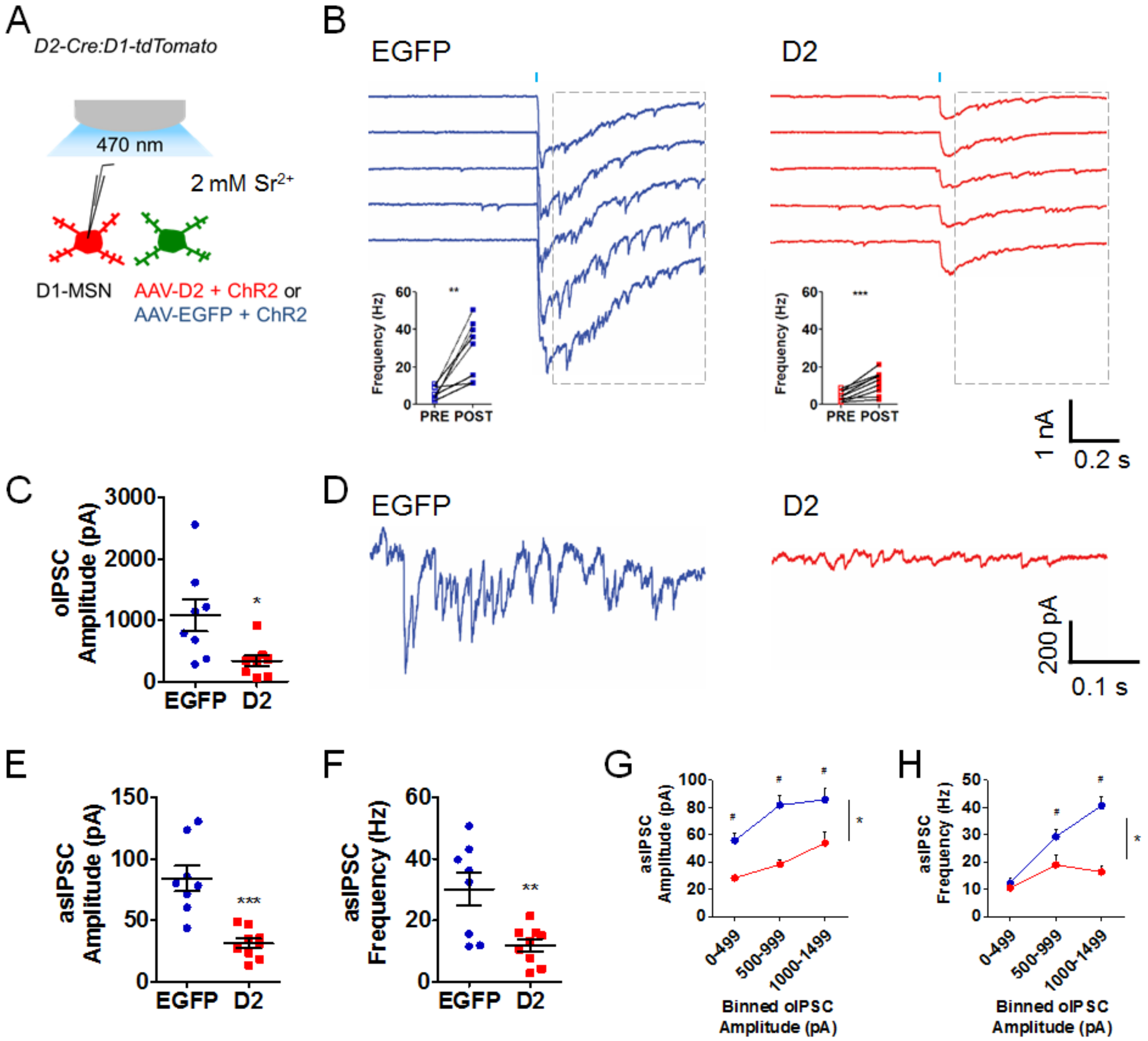

Supplementary Figure 6. D2R upregulation in D2-MSNs leads to decreased asynchronous IPSC frequency and amplitude in D1-MSNs. ***A.*** Schematic illustrating whole-cell recordings from tdTomato-positive, D1-containing MSNs during photostimulation of D2-MSNs expressing either D2 or EGFP in the presence of 2 mM strontium. ***B***. Representative traces from one cell per group, showing that 5 ms photostimulation led to an initial synchronous optically-evoked IPSC (oIPSC), followed by delayed, smaller amplitude currents (asIPSCs) in both groups. *Insets*, frequency of the inhibitory events following light stimulation (dotted box) was higher than in a prestimulus period of the same duration in all cells from both groups [EGFP: $t = 4.519$, p = 0.003, 8 (4) cells; D2: $t = 5.272$, p = 0.001, 9 (2) cells], verifying that asIPSCs were successfully triggered by photostimulation. ***C.*** Initial synchronous oIPSC amplitude was significantly reduced in D2R-OE_{NAcInd} mice compared to controls ($t = 2.826$, p =0.013, 8 (4) or 9 (2) cells/group). ***D.*** Enlarged view of the asIPSC analysis period (50-500 ms after stimulus) for the bottommost trace for each

group in ***B***, showing reduced amplitude and frequency of asIPSCs in D2R-OE_{NAcInd} mice. ***E.*** Mean asIPSC amplitude was significantly decreased following D2R upregulation in D2-MSNs (t = 4.975, p = 0.0002, 8 (4) or 9 (2) cells/group). ***F.*** Mean asIPSC frequency was also reduced in D2R-OE_{NAcInd} mice (t = 3.323, p = 0.005, 8 (4) or 9 (2) cells/group) ***G, H.*** To normalize asIPSC amplitude and frequency to the initial stimulus strength, asIPSCs from individual trials were binned according to the size of their associated oIPSCs. Compared to controls, D2R-OE_{NAcInd} mice consistently showed smaller asIPSC amplitude than controls despite comparable oIPSC size ($F_{(1,156)} = 38.64$, $p < 0.0001$). asIPSC frequency was similar between both groups when the initial oIPSC amplitudes for both groups were less than 500 pA. At equivalent initial oIPSC amplitudes above 500 pA, however, asIPSC frequency was significantly decreased in D2R-OE_{NAcInd} mice (virus x oIPSC amplitude interaction: $F_{(2,152)} = 8.342$, $p < 0.0005$), suggesting a dissociation between large initial release events and asIPSC frequency in the D2R-OE_{NAcInd} mice. Error bars = s.e.m.

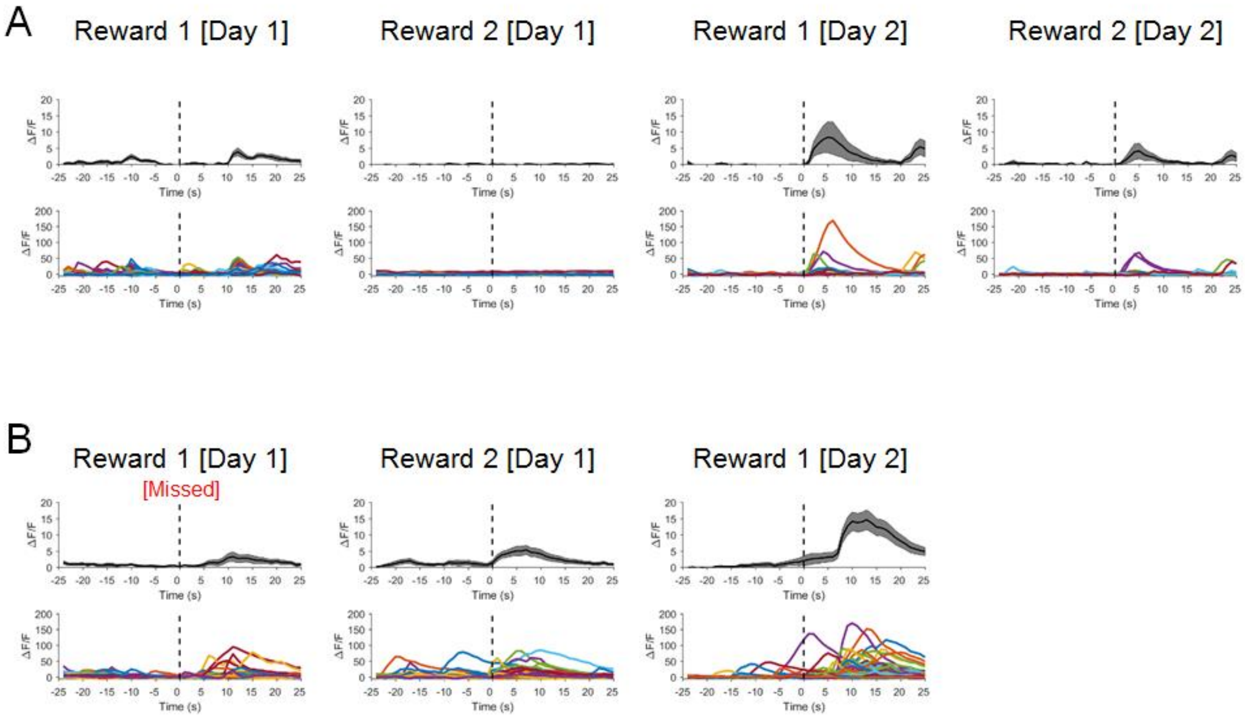

Supplementary Figure 7. Calcium activity associated with reward retrieval. Examples of calcium activity measured 5 sec before and after rewarded head entries (red dotted line) over 2 days of PR. In each example, the top panels show the average Ca^{2+} response of all cells, while the bottom panels show the response of each individual cell. ***A.*** This mouse exhibits a variable Ca^{2+} response across each reward obtained, with only a few cells showing a distinct response on Day 2. ***B.*** This mouse showed a more robust Ca^{2+} response associated with reward retrieval, but also exhibited similar Ca^{2+} responses when it missed a reward. Similar responses were observed in 3 mice per group.

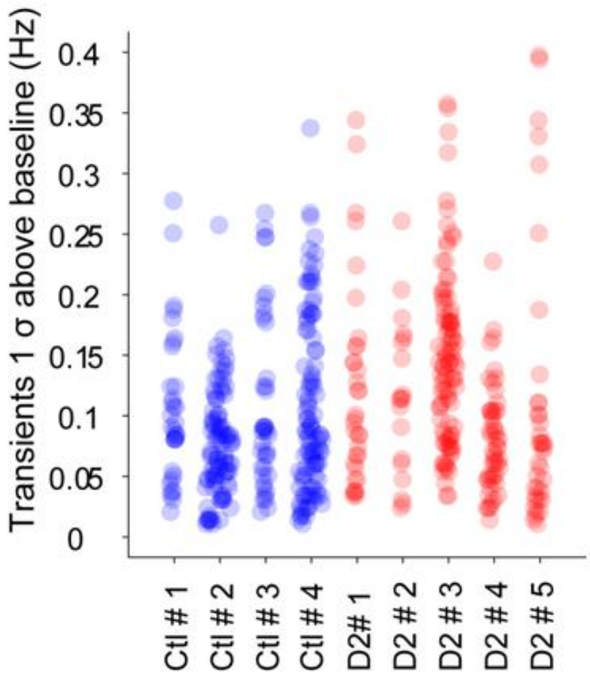

Supplementary Figure 8. D1-MSN activity is not altered during a fixed ratio schedule. Transient frequency during FR5 task for each cell analyzed in control (n = 4 mice) and in D2R-OE_{NAcInd} mice (n = 5 mice).

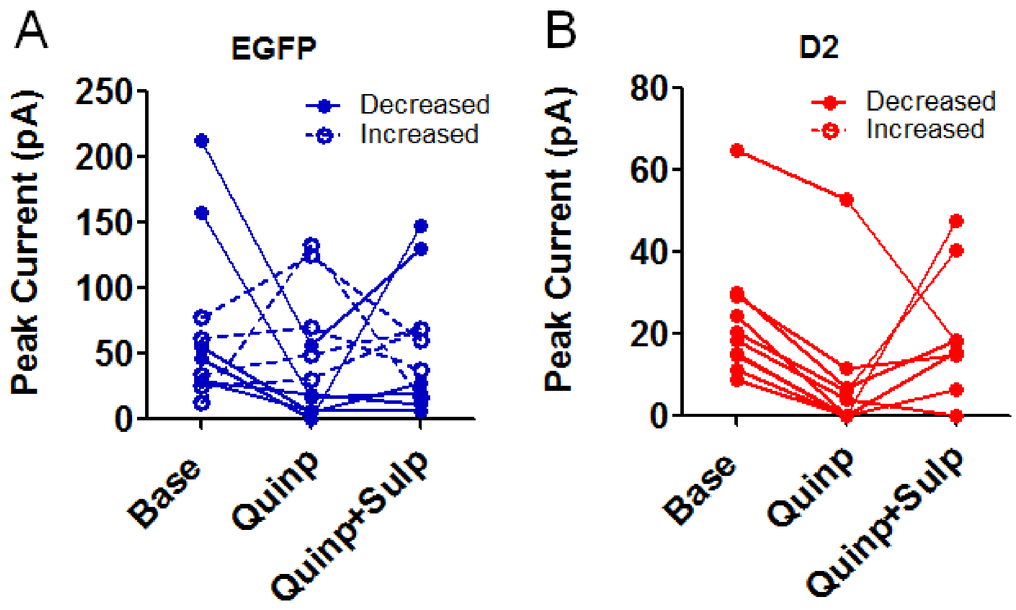

Supplementary Figure 9. Effects of quinpirole and sulpiride on oIPSCs measured in VP neurons. Response plots of individual neurons in Figure 4*G* from $EGFP_{NAcInd}$ mice ***A.*** and D2R-OE_{NAcInd} mice ***B.*** showing the effect of quinpirole on oIPSC amplitude. n = 11 neurons/group. In control $EGFP_{NAcInd}$ VP neurons, quinpirole either decreased or increased the oIPSC, while it predominantly decreased oIPSCs in D2R-OE_{NAcInd}.

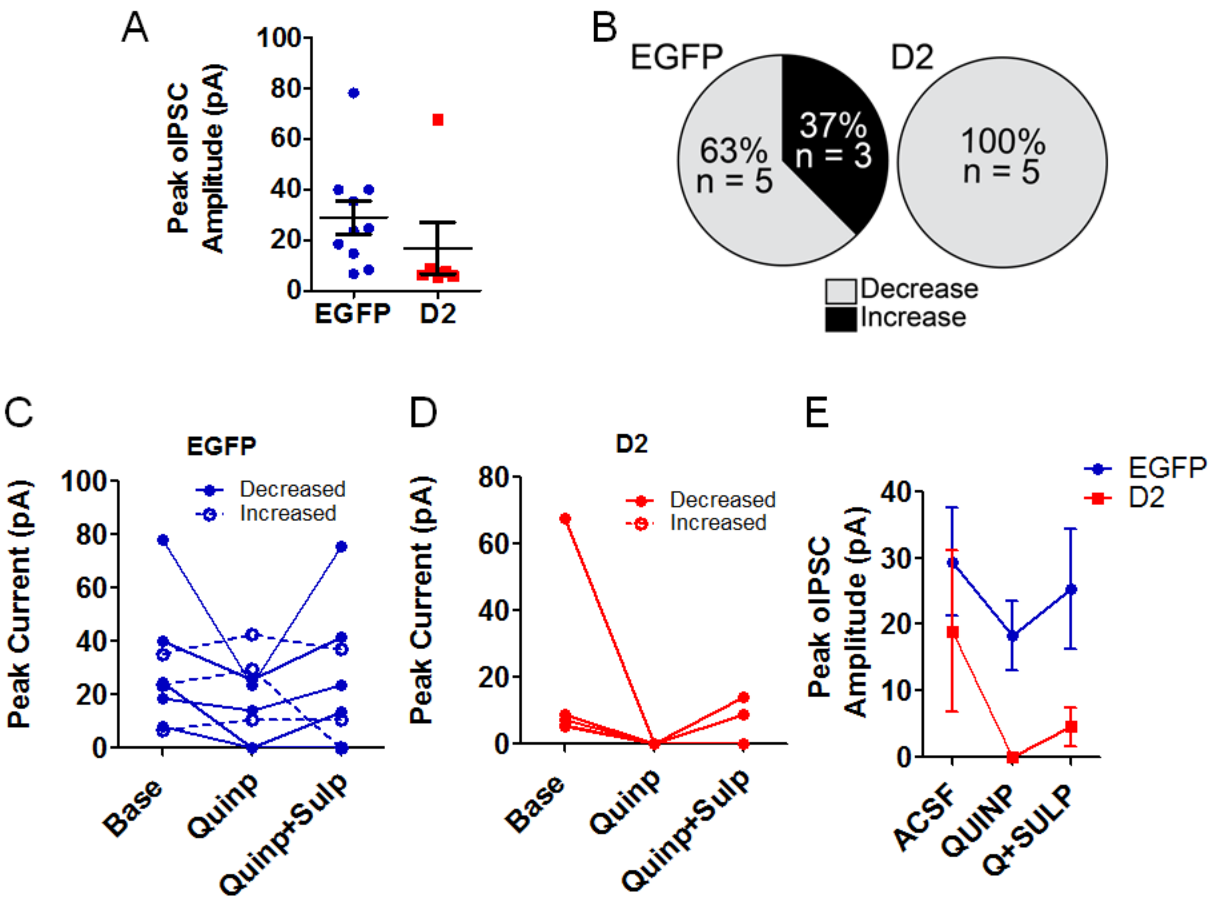

Supplementary Figure 10. Effects of D2R upregulation on oIPSC amplitude in VP neurons are observable with short-duration stimulation. ***A.*** Effects of D2R upregulation on the peak amplitude of oIPSCs evoked 1-ms pulses ($p > 0.05$, EGFP, n = 10 (8) and D2, n = 6 (6) cells/group). ***B.*** Quinpirole treatment led to a reduction in oIPSC size in 63% and 100% of VP neurons recorded in $EGFP_{NAcInd}$ and in $D2R_{NAcInd}$ mice, respectively. ***C, D.*** Individual neuron responses to quinpirole followed by quinpirole + sulpiride in both groups. ***E.*** Averaged responses to neurons shown in *C, D*. Error bars = s.e.m.

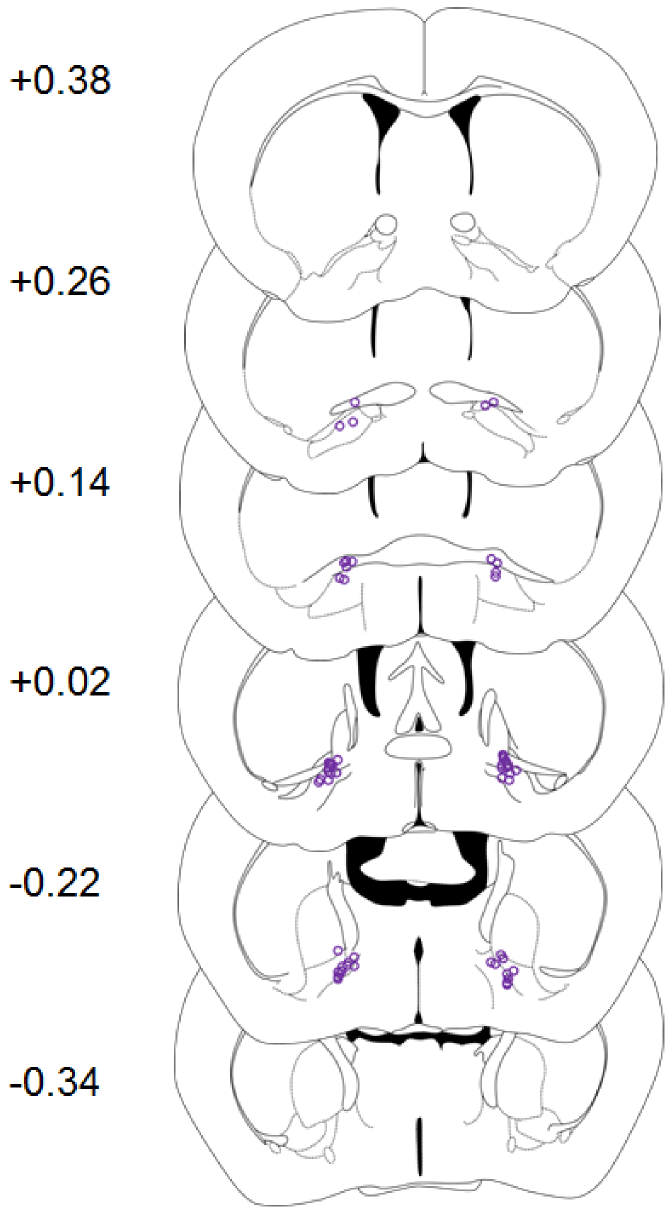

Supplementary Figure 11. Cannula targeting of the VP. Schematic representation of the location of cannula tips targeting the caudal VP in mice included in the behavioral analysis. Coronal planes depicted are based on Franklin and Paxinos. *The Mouse Brain in Stereotaxic Coordinates*, 3rd edition. (Academic Press, New York, 2007).

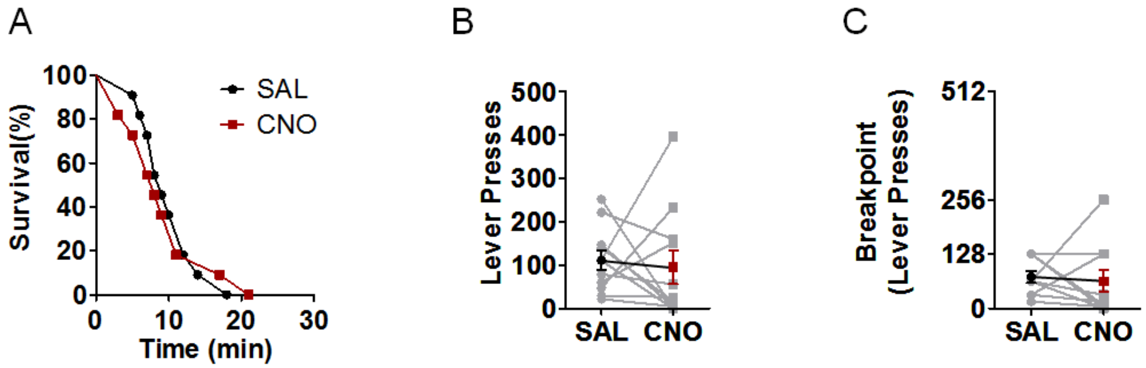

Supplementary Figure 12. CNO does not alter motivation in the absence of hM4Di. Four weeks after surgery, CNO (1 mM) or saline was delivered (0.3 µl/side) to the caudal VP prior to PR testing session. All 12 mice expressing either EGFP or no virus received both treatments on different days in counterbalanced fashion. CNO did not significantly alter PR responding, as shown in the session duration plotted as a survival function (***A***), total lever presses (***B***), and breakpoint (***C***).
